# Perspective from a general hospital psychiatric unit

**DOI:** 10.4103/0019-5545.43053

**Published:** 2005

**Authors:** Smita N. Deshpande

**Affiliations:** Senior Psychiatrist and Head

There is no doubt that mental disorders are expensive. They incur huge costs—including direct (expenditure) as well as indirect (loss of income) costs.^1^ But policy-makers ask for proof, which is as yet scarce in India. Besides, changing economic scenarios—opening up of the Indian economy, changing patent laws (of special concern for consumers and pharmaceutical companies alike), the World Trade Organization Agreements^2^—may make old observations about expenditure lesser than at present and sometimes even redundant. Nevertheless, some preliminary observations are presented below.

Using World Health Organization (WHO) funds, we completed a study in 2002–03 to train families in the rehabilitation and management of their ill relative. One of the components was evaluation of the costs incurred for the treatment of individuals with schizophrenia (SZ, *n*=97) and bipolar disorder (BPD, *n*=25). We surmised that the costs of travel for care, time taken off from work and related expenses were quantifiable costs that had not been measured. Given the small number of psychiatrists and inadequate exposure of medical students to Psychiatry (leading to inability of primary care physicians to offer mental health care), travel costs could be quite high. Thus, most hospitals in Delhi cater to a large number of patients residing in the surrounding states where psychiatric coverage is insufficient.

Following Murthy (personal communication),^3^ Chisholm^4^ and Patel *et al*.,^5^ we tried to calculate the direct, indirect and intangible costs of mental health care for the three months immediately prior to the research interview during the last quarter of 2002. Costs were calculated for both the participants and their caregivers, since both groups of individuals were expected to attend for treatment sessions. The costs included travel, consultation fees (since Ram Manohar Lohia Hospital [RMLH] is a free government facility, this was calculated for other physicians or faith healers). As drugs are dispensed free at RMLH, we calculated drug costs from the *Drug Index 2002* or local pharmacies. Thus, direct costs were estimated. For indirect costs, we estimated the monetary loss by time lost during visits to treatment facilities as well as time taken for relatives to look after patients and to carry out the patient's duties (i.e. two individuals per patient).

While 78% of our participants were less than 45 years of age, 55% of caregivers were more than 45 years of age, adding to the latter's physical burden. In many cases, aged parents (50% of cases) or spouses (36%) looked after their ill relative. In most cases, the caregivers were old mothers or ageing wives. The patients were more educated than their caregivers (13% of the patients had received primary education and 42% secondary education; 31% of caregivers had received primary education and 37% of them secondary education). By and large, the patients of SZ were unable to seek or maintain jobs, look after their personal hygiene, ensure their own nutrition, retain social relationships or even use public transport. Almost half had children they themselves were unable to care for. Estimates were very similar for BPD patients and their caregivers too.

Among the patients attending RMLH, 6 out of 95 individuals with SZ and 3 out of 25 patients with BPD had consulted traditional healers during the past three months. Ten patients with SZ and 2 with BPD had consulted primary care physicians during the past three months. The fees paid to these facilities were very high.

In contrast with SZ patients, BPD patients paid dis-proportionately higher fees for consultations with traditional healers (between Rs 400 and 1000 for SZ patients vs Rs 300–6000 for patients with BPD). While SZ patients spent Rs 400–1000 on travel to these non-psychiatric facilities, BPD patients spent Rs 1500–3000. Primary care costs were in the intermediate range (between traditional healers and RMLH) of these expenditures. Higher travel costs also imply more time spent in travel.

Medical costs were also high. We calculated these costs even if a patient had received drugs free at RMLH. Though free for the patient, these costs were borne by the tax-paying public. Even when patients required only one drug, costs varied per month from Rs 288±232 for SZ to Rs 364±294 for BPD. Considering that these costs could go on indefinitely and could increase during relapses, these are alarming figures.

Loss of time for caregivers was also high—caregivers who were housewives spent 96 hours per month (SZ) or 153 hours per month (BPD) on average looking after the patient or taking over his/her duties, while working caregivers spent 109 hours/month for SZ and 84 hours/month for BPD patients. If a second caregiver was also involved, there was further loss of time: 60 hours/month for SZ and 34 hours/month for BPD. Though 9 patients were reportedly looking after themselves, their level of self-care was uncertain.

Overall, families of BPD patients spent more time and money than those of SZ patients. This may be an artifact because we had recruited families attending the outpatient clinic—stable SZ patients may have attended less often and may have required less medication. Since stable outpatients on only one medication were included, the artifact could also be explained on the basis of an increased likelihood of BPD patients being prescribed more expensive mood stabilizers.

Other Indian researchers focused on cost issues. Grover *et al.*^6^ emphasized the high cost of SZ treatment. These workers found that the costs of SZ treatment were comparable with those for the treatment of diabetes, but felt that indirect costs were higher than direct costs. Their estimate for 6-month expenses was US$ 274. More severely ill individuals needed to attend hospital clinics more often and therefore incurred higher costs.

Female patients may be perceived to incur higher costs due to their lack of earning capacity and the need for families to care for their offspring.^7^ Murthy *et al.*^8^ showed that care for patients at their doorsteps and/or in their communities decreased costs progressively over time.

What are the remedies for these relatively high costs? Obviously, the ultimate remedy is a cure. This remains a distant goal.

What should the community do to reduce costs and the time taken for provision of care? To some extent, the following measures are necessary:

- Bring medicines to the patients' doorsteps, so that time and expenditure for travel can be reduced.^8^
- Try to keep medication costs low by modifying relevant laws.
- Doctors should try to prescribe fewer medications at optimal doses.
- Patients and families should try to prevent relapse by regular medication, primarily to contain hospital costs as they are expensive in terms of money, time and emotional costs.
